# Supplementary material for: Superior efficacy of combination antibiotic therapy versus monotherapy in a mouse model of Lyme disease
Source: Front Microbiol. 2023 Nov 21;14:1293300. doi: 10.3389/fmicb.2023.1293300 (PMC10703379; doi:10.3389/fmicb.2023.1293300)
Supplement: Supplementary file 1 [file Data_Sheet_1.docx]

**Supplementary Materials**

**Table S1. Primers Used for Molecular Detection.**

| Primer Name | | Sequence | Reference |
| --- | --- | --- | --- |
| *16S* | **Reverse** | **5’-GGC GGC CAC TTA ACA CGT TAG-3’** | **(Ornstein & Barbour, 2006)** |
|  | **Forward** | **5’-GGTCAAGACTGA CGC TGA GTC A-3’** |  |
| *OspC* | **Reverse** | **5’- CAT ACT CGA GTT AAG GTT TTT TTG GAC-3’** | **(Embers et al., 2008)** |
|  | **Forward** | **5’- CAT AGG ATC CAA TTC AGG GAA AGA TG-3’** |  |
| *OspA* | **Reverse** | **5′-TCGTACTTGCCGTCTTTGTT -3′** | **(Embers et al., 2012)** |
|  | **Forward** | **5′-AATGTTAGCAGCCTTGACGA-3′** |  |

**Table S2: FDA-approved Drugs effective against Bb in vitro.**

| Drug Name (Class) | Mechanism of Action | In vitro (*Bb* Culture) | | References |
| --- | --- | --- | --- | --- |
|  |  | MIC | MBC |  |
| Azlocillin  (Penicillin) | Binds to specific penicillin-binding proteins (PBPs) inside the bacterial cell wall. It also Inhibits the third and last stages of the synthesis of the bacterial cell wall where the autolytic enzymes of the bacterial cell wall take place. | 1.5 (μM) | 2.5 (μM) | (DrugBank, 2021a; Pothineni et al., 2016; Ulrich et al., 1989) |
| Bactrim  (Sulfonamides/ Trimethoprim) | Blocks folic acid synthesis. Sulfonamides (S) Inhibits the enzymes involved in the production of dihydrofolic acid and tetrahydrofolic acid. Trimethoprim(T) Inhibits the production of tetrahydrofolic acid related enzymes. | 0.4 μg/mL | >200 μg/mL (failed to completely eradicate *Bb* even at 200 μg/mL) | (Jie Feng et al., 2017; Wróbel et al., 2020) |
| Carbomycin (Macrolides) | A macrolide inhibits the synthesis of protein by targeting the 50S ribosomal subunit. It also blocks the formation of peptide bonds between amino acids. | ≤0.25 μg/mL | Bacteriostatic, no MBC is expected | (Jie Feng et al., 2014; Martens & Demain, 2017) |
| Cefotaxime  (Third Generation Cephalosporins) | Injectable Semisynthetic cephalosporin that inhibits transpeptidases activity of penicillin-binding proteins. It is also a broad-spectrum antibiotic that works against gram-positive and gram-negative bacteria, including some β-lactamase producers. | ≤3 µM ≤0.03 μg/mL | ≤10 µM 0.25 μg/mL | (DrugBank, 2021b; Levin et al., 1993; Pothineni et al., 2016) |
| Ceftriaxone  (Third Generation Cephalosporins) | Injectable bactericidal cephalosporin that inhibits transpeptidases activity of penicillin-binding proteins (PBPs) which leads to inhibition of cell wall synthesis. Based on its adverse effect it is considered safe with a long half-life. | 0.03 μg/mL | 0.06 μg/mL | (DrugBank, 2021c; Sicklinger et al., 2003) |
| Clofazimine (Riminophenazine dye, antimycobacterial) | Block the template function of the bacterial DNA and inhibit its proliferation by binding to the guanine bases of the DNA. The riminophenazine dye is soluble in fat and is used for the treatment of leprosy. It is also used in combination with other antimycobacterial drugs to treat *Mycobacterium avium* infections in AIDS patients. | 6.25 μg/mL | NA | (DrugBank, 2021d; Jie Feng et al., 2014) |
| Dapsone (Sulfones) | Inhibits bacterial dihydrofolic acid synthesis. Inhibits bacterial synthesis by inhibiting the production of dihydrofolic acid enzyme. It is a broad-spectrum antibiotic against gram-positive and gram-negative bacteria. it commonly used as the treatment of leprosy with a combination of rifampicin and clofazimine. | 0.4 μg/mL | >200 μg/mL (failed to completely eradicate *Bb* even at 200 μg/mL) | (DrugBank, 2021e; Jie Feng et al., 2017) |
| Disulfiram (Disulfide/Antabuse) | A carbamate derivative that can penetrate the cell wall and inhibiting aldehyde dehydrogenase. It is used as an alcohol deterrent. | 0.625 (μM) | 1.25 (μM) | (DrugBank, 2021f; Liegner, 2019; Pothineni et al., 2016) |
| Doxycycline (Tetracyclines) | Broad-spectrum, bacteriostatic, antibiotic that inhibits translation and protein synthesis by blocking the association of tRNAs with the ribosome during translation. | 0.25 - 0.5 μg/mL | 8 μg/mL | (DrugBank, 2021g; Embers et al., 2013; Jie Feng et al., 2017; Sicklinger et al., 2003) |
| Loratadine (Second Generation Antihistamine) | Second generation antihistamine. selective for peripheral H1 receptors. Loratadine does not penetrate effectively into the central nervous system and has a poor affinity for CNS H1-receptors. These qualities result in a lack of CNS depressant effects such as drowsiness, sedation, and impaired psychomotor function. It can also block Mn transport in *Borrelia burgdorferi*. The elimination half-life is approximately 10 hours. | >77.7 μg/mL | >77.7 μg/mL | (DrugBank, 2021h; Wagh et al., 2015) |
| Rifampicin  (Rifamycin) | Narrow spectrum antibiotic that is active against gram-positive and some of the gram-negative bacteria. It is commonly used in combination therapy with dapsone and clofazimine for the treatment of TB. It works by inhibiting the DNA-dependent RNA polymerase activity in the bacterial cell without affecting the mammalian enzyme. | 0.4 μg/mL | >200 μg/mL (failed to completely eradicate *Bb* even at 200 μg/mL) | (DrugBank, 2021i; Jie Feng et al., 2017) |

**Table S3: Infection confirmation for mice in the monotherapy experiment**

| Treatments | Infection Confirmation | | |
| --- | --- | --- | --- |
|  | **Ear Biopsy^*** | **Serology (5-Ag Test)^§** | **Total Number of Mice Infected^** |
| **Infected Untreated (OG)** | 0/3 | 3/3 | 3/3 |
| **Infected Untreated (IP)** | 3/5 | 5/5 | 5/5 |
| **Infected Untreated (SC)** | 1/2 | 2/2 | 2/2 |
| **Azlocillin (IP)** | 2/5 | 5/5 | 5/5 |
| **Bactrim (OG)** | 1/5 | 5/5 | 5/5 |
| **Disulfiram (OG)** | 1/5 | 5/5 | 5/5 |
| **Carbomycin (SC)** | 2/5 | 5/5 | 5/5 |
| **Dapsone (OG)** | 0/5 | 5/5 | 5/5 |
| **Rifampicin (OG)** | 2/5 | 5/5 | 5/5 |
| **Cefotaxime (IP)** | 4/5 | 5/5 | 5/5 |
| **Loratadine (OG)** | 4/5 | 5/5 | 5/5 |
| Mouse infection tested in 2 different methods: Ear biopsy and serology, and combined results.  ^Number of mouse positive / total number of mice tested.  *At week 1 or 2 post infection.  **§** at least 2 Antigens or more were positive at day 21 or 60 post infection. OG= oral gavage, IP= intraperitoneal, SC= subcutaneous | | | |

**Table S4: Summary of the MKB results showing the zone of inhibition of drugs in pooled sera.**

| Day of Treatment | Drug | Number of Sample (Pooled Sera of 5 Mice per Group, Total of 25μl per Sample) | Diameter Reads of Zone of Inhibition (mm) | | | | |
| --- | --- | --- | --- | --- | --- | --- | --- |
|  |  |  | **Read 1** | **Read 2** | **Read 3** | **Average of 3 Reads** | **Average of the 2 Samples After Averaging the 3 Reads** |
| Day 66 | **Rifampicin** | **1** | 12.5 | 13 | 13 | 12.83 | 13 |
|  |  | **2** | 13.5 | 13 | 13 | 13.17 |  |
|  | **Cefotaxime** | **1** | 2.5 | 2.5 | 2 | 2.33 | 1.17 |
|  |  | **2** | 0 | 0 | 0 | 0 |  |
|  | **Carbomycin** | **1** | ND | ND | ND | ND | ND |
|  |  | **2** | ND | ND | ND | ND |  |
|  | **Control Untreated Mice** | **1** | 0 | 0 | 0 | 0 | 0 |
|  |  | **2** | 0 | 0 | 0 | 0 |  |
| Day 73 | **Rifampicin** | **1** | 12 | 11 | 11 | 11.33 | 11.33 |
|  |  | **2** | 12 | 11 | 11 | 11.33 |  |
|  | **Cefotaxime** | **1** | ND | ND | ND | ND | ND |
|  |  | **2** | ND | ND | ND | ND |  |
|  | **Carbomycin** | **1** | 2.5 | 2 | 2 | 2.17 | 2.33 |
|  |  | **2** | 2.5 | 2.5 | 2.5 | 2.50 |  |
|  | **Control Untreated Mice** | **1** | 0 | 0 | 0 | 0 | 0 |
|  |  | **2** | 0 | 0 | 0 | 0 |  |
| Day 80 | **Rifampicin** | **1** | 9 | 9 | 10 | 9.33 | 9.33 |
|  |  | **2** | 9 | 9 | 10 | 9.33 |  |
|  | **Cefotaxime** | **1** | N/A | N/A | N/A | N/A | N/A |
|  |  | **2** | N/A | N/A | N/A | N/A |  |
|  | **Carbomycin** | **1** | N/A | N/A | N/A | N/A | N/A |
|  |  | **2** | N/A | N/A | N/A | N/A |  |
|  | **Control Untreated Mice** | **1** | 0 | 0 | 0 | 0 | 0 |
|  |  | **2** | 0 | 0 | 0 | 0 |  |

| ND= not done. N/A= samples not available. |
| --- |

**Table S5: Determination of rifampicin concentration in mouse serum by MKB.**

| Rifampicin Serial Dilution | Concentration (μg/ml) | Zone of Inhibition (Diameter in mm) |  |
| --- | --- | --- | --- |
| 1 | 10 | 20.17 |  |
| 2 | 5 | 16.83 |  |
| 3 | 1 | 11.33 |  |
| 4 | 0.5 | 8.83 |  |
| 5 | 0.25 | 7 |  |
| 6 | 0.1 | 5 |  |
| Rifampicin Day 66 of Treatment | **Sample 1** | **Sample2** | **Average Samples** |
| Zone of Inhibition (Diameter in mm) | 12.83 | 13.17 | 13 |
| Drug Concentration (μg/ml) Based on the Standard Curve | 3.75 | 3.99 | 3.87 |
| Rifampicin Day 73 of Treatment | **Sample 1** | **Sample2** | **Average Samples** |
| Zone of Inhibition (Diameter in mm) | 11.33 | 11.33 | 11.33 |
| Drug Concentration (μg/ml) Based on the Standard Curve | 2.67 | 2.67 | 2.67 |
| Rifampicin Day 80 of Treatment | **Sample 1** | **Sample2** | **Average Samples** |
| Zone of Inhibition (Diameter in mm) | 9.33 | 9.33 | 9.33 |
| Drug Concentration (μg/ml) Based on the Standard Curve | 1.23 | 1.23 | 1.23 |
| Average of Zone of Inhibition of All 3 Days of Treatment for All Samples (mm) | 11.22 | | |
| Drug Concentration (μg/ml) For All Treatment Days. Based on the Standard Curve | 2.59 | | |

**Figure S1: Rifampicin standard curve based on the serial dilution of the drug for determination of sample concentration.**

The x-axis represents the drug concentrations (μg/ml). The y-axis represents the zone of inhibition (diameter in mm).

**Table S6: Determination of cefotaxime concentration in mouse serum by MKB.**

| Cefotaxime Serial Dilution | Concentration (μg/ml) | Zone of Inhibition (Diameter in mm) |  |
| --- | --- | --- | --- |
| 1 | 10 | 3.83 |  |
| 2 | 5 | 2.67 |  |
| 3 | 1 | 0 |  |
| 4 | 0.5 | 0 |  |
| 5 | 0.25 | 0 |  |
| Cefotaxime Day 66 of Treatment | **Sample 1** | **Sample2** | **Average Samples** |
| Zone of Inhibition (Diameter in mm) | 2.33 | 0 | 1.17 |
| Drug Concentration (μg/ml) Based on the Standard Curve | 5.18 | 0 | 2.45 |

**Figure S2: Cefotaxime standard curve based on the serial dilution of the drug for determination of sample concentration.**

The x-axis represents the drug concentrations (μg/ml). The y-axis represents the zone of inhibition (diameter in mm).

**Table S7: Determination of carbomycin concentration in mouse serum by MKB.**

| Carbomycin Serial Dilution | Concentration (μg/ml) | Zone of Inhibition (Diameter in mm) |  |
| --- | --- | --- | --- |
| 1 | 10 | 4 |  |
| 2 | 5 | 3.5 |  |
| 3 | 1 | 1.083 |  |
| 4 | 0.5 | 0 |  |
| 5 | 0.25 | 0 |  |
| Carbomycin Day 73 of Treatment | **Sample 1** | **Sample2** | **Average Samples** |
| Zone of Inhibition (Diameter in mm) | 2.17 | 2.5 | 2.33 |
| Drug Concentration (μg/ml) Based on the Standard Curve | 4.42 | 5.19 | 4.79 |

**Figure S3: Carbomycin standard curve based on the serial dilution of the drug for determination of sample concentration.**

The x-axis represents the drug concentrations (μg/ml). The y-axis represents the zone of inhibition (diameter in mm).

**Table S8: Result of the PCR/ RT-PCR for xenodiagnosis for monotherapy**

| Treatments | PCR (DNA) | | RT-PCR (RNA) | |
| --- | --- | --- | --- | --- |
|  | ***16S^^^*** | ***ospA^^^*** | ***16S^^^*** | ***ospA^^^*** |
| Infected Untreated (OG) | 3/3 | 3/3 | 3/3 | 3/3 |
| Infected Untreated (IP) | 3/5 | 2/5 | 5/5 | 5/5 |
| Infected Untreated (SC) | 0/2 | 0/2 | 1/2 | 2/2 |
| Azlocillin (IP) | 4/5 | 3/5 | 4/5 | 4/5 |
| Bactrim (OG) | 3/5 | 4/5 | 5/5 | 3/5 |
| Disulfiram (OG) | 2/5 | 4/5 | 5/5 | 5/5 |
| Carbomycin (SC) | 4/5 | 2/5 | 5/5 | 3/5 |
| Dapsone (OG) | 1/5 | 2/5 | 3/5 | 3/5 |
| Rifampicin (OG) | 1/5 | 0/5 | 2/5 | 2/5 |
| Cefotaxime (IP) | 1/5 | 2/5 | 1/5 | 1/5 |
| Loratadine (OG) | 2/5 | 2/5 | 2/5 | 3/5 |
| Pooled xenodiagnostic ticks per mouse tested by different molecular detection methods: PCR and RT-PCR for using 2 primers *ospA* and *16S*.  ^ Number of pooled ticks per mouse positive / total number of mice tested. OG= oral gavage, IP= intraperitoneal, SC= subcutaneous. | | | | |

**Table S9: Infection confirmation for mice in the combination experiment**

| *Drugs* | Infection Confirmation | | |
| --- | --- | --- | --- |
|  | **Ear Biopsy^*** | **Serology (5-Ag Test)^§** | **Total Number of Mice Infected^** |
| **Infected Untreated (OG)** | 0/3 | 3/3 | 3/3 |
| **Infected Untreated (IP)** | 3/5 | 5/5 | 5/5 |
| **Infected Untreated (SC)** | 1/2 | 2/2 | 2/2 |
| **Azlocillin (IP) + Bactrim (OG)** | 3/5 | 5/5 | 5/5 |
| **Disulfiram (OG) + Bactrim (OG)** | 2/5 | 5/5 | 5/5 |
| **Disulfiram (OG) + Azlocillin (IP)** | 4/5 | 5/5 | 5/5 |
| **Doxycycline (OG) + Ceftriaxone (IP)** | 4/5 | 5/5 | 5/5 |
| **Doxycycline (OG) + Ceftriaxone (IP) + Carbomycin (SC)** | 4/5 | 5/5 | 5/5 |
| **Dapsone (OG) + Rifampicin (OG)** | 0/5 | 5/5 | 5/5 |
| **Dapsone (OG) + Clofazimine (OG)** | 0/5 | 5/5 | 5/5 |
| **Dapsone (OG) + Clofazimine (OG) + Rifampicin (OG)** | 0/5 | 5/5 | 5/5 |
| **Cefotaxime (IP) + Loratadine (OG) + Doxycycline (OG)** | 2/5 | 5/5 | 5/5 |
| **Cefotaxime (IP) + Doxycycline (OG)** | 2/5 | 5/5 | 5/5 |
| **Cefotaxime (IP) + Carbomycin (SC)** | 2/5 | 5/5 | 5/5 |
| Mouse infection tested in 2 different methods: Ear biopsy and serology, and combined results.  ^Number of mouse positive / total number of mice tested.  *At week 1 or 2 post infection.  § at least 2 Antigens or more were positive at day 21 or 60 post infection. OG= oral gavage, IP= intraperitoneal, SC= subcutaneous | | | |
